# On Cox proportional hazards model performance under different sampling schemes

**DOI:** 10.1371/journal.pone.0278700

**Published:** 2023-04-26

**Authors:** Hani Samawi, Lili Yu, JingJing Yin

**Affiliations:** Department of Biostatistics, Epidemiology and Environmental Health Sciences, Jiann-Ping Hsu College of Public Health, Georgia Southern University, Statesboro, Georgia, United States of America; Amity University - Lucknow Campus, INDIA

## Abstract

Cox’s proportional hazards model (PH) is an acceptable model for survival data analysis. This work investigates PH models’ performance under different efficient sampling schemes for analyzing time to event data (survival data). We will compare a modified Extreme, and Double Extreme Ranked Set Sampling (ERSS, and DERSS) schemes with a simple random sampling scheme. Observations are assumed to be selected based on an easy-to-evaluate baseline available variable associated with the survival time. Through intensive simulations, we show that these modified approaches (ERSS and DERSS) provide more powerful testing procedures and more efficient estimates of hazard ratio than those based on simple random sampling (SRS). We also showed theoretically that Fisher’s information for DERSS is higher than that of ERSS, and ERSS is higher than SRS. We used the SEER Incidence Data for illustration. Our proposed methods are cost saving sampling schemes.

## Introduction

The survival time of a particular event is called the time-to-event. The time of death and time to develop a disease are examples of survival data. Statistical methods for survival analysis have been applied to many vital fields of research. Generally, survival analysis uses data to predict survival probability and identify risk and/or prognostic factors related to subjects’ survival and disease progression. An essential aspect of survival data is not usually fully observed in all subjects under study, leading to different censored data types.

Subjects in a study are usually assumed to be selected randomly (interred the study randomly) in the sense of simple random sample (SRS) [1]. Another promising sampling method in the literature is Ranked set sampling (RSS) introduced by McIntyre [2, 3]. RSS is a useful cost affected sampling scheme for agriculture and environmental studies. Samawi and Al-Sagheer [4] applied RSS in a study involving human subjects by collecting data in the study based on bilirubin’s level in the jaundice premature babies’ blood. Also, recently Jabrah et al. [5] used RSS in a study involving human subjects, and they selected college students to analyze a psychological intervention to buttress resilience study. More applications of RSS and variations of RSS and their efficiency for estimation population means and other parameters are presents recently by several authors such as [6–14] among others.

RSS (Balanced) is collected by drawing *m*^*2*^ items randomly from a target population and then randomly dividing them into *m* sets of *m* units. Units from each set of size m then ranked virtually, or by the mean of the available auxiliary variable (concomitant) that associated with the variable of interest. Among the first set of *m* units, we choose the unit with the lowest rank and measure the unit’s actual value. In the second set unit with the second-lowest ranking is measured, and so on until the unit with the highest ranking is measured from the last set. It is essential to point out that the ranking error will reduce RSS’s efficiency compared with SRS in some inferential procedures. Therefore, it is crucial to choose a ranking method that improves the ranking precision [15, 16].

Furthermore, when the set size *m* is large, selecting an RSS sample of size *m* is susceptible to ranking errors, so the literature suggested m not to excide 5. However, to overcome size r of m restriction to reduce ranking errors, several modifications and variations of the RSS are suggested in the literature. Samawi et al. [17] were the first to introduce the ERSS sampling scheme, while Al-Odat and Al-Saleh [18] proposed moving Extreme Ranked Set Sampling (MERSS). Al-Saleh and Samawi [19] and Samawi and Al-Saleh [20] implemented both MERSS_max_ and MERSS_min_ to provide valid odds and more efficient estimates of the odds ratio, respectively. Samawi et al. [21] investigates the use of MERSS_min (max)_ to improve the Cox proportional hazards model’s efficiency. This modified sample’s advantage is only the maximum (or minimum) sets of varied sizes are identified for quantification in this procedure. Therefore, even for large m, MERSS, or ERSS, can be easily implemented.

As another modification of RSS, is the Double Extreme Ranked Set Sampling (DERSS) sampling scheme was introduced by Samawi [22] for the mean and regression estimators. Helu et al. [23] improved the AFT survival model’s analysis efficiency using a modified version of ERSS, namely ERSSmin (ERSSmax). Also, Samawi et al. [24] used the modified MERSS to improve the logistic regression analyses’ performance. Recently, Samawi et al. [25] further enhances logistic regression analysis using modified DERSS.

The DERSS_min_ (DERSS_max_) procedure, which is an extension to ERSS procedure, is suggested here. This procedure involves randomly drawing *m* independent sets each contains *m* random samples of size *m* each (note that each set has *m*^*2*^ sample units). Each sample is drawn from a large population. We assume that the largest or the smallest sample unit within each sample with respect to the value of an auxiliary variable Z, which is associated with survival time, can be identified with no or little cost. From each set of size *m*^*2*^ use ERSS procedure, as described by Samawi at el. [17] to obtain *m* ERSS_min_ (or ERSS_max_) samples of size *m* each (this completes the first stage). Again apply the same ERSS_min_ (or ERSS_max_) procedure on the obtained *m* ERSS_min_ (or ERSS_max_) samples of size *m* each to obtain a sample of size *m*, which will be called the second stage of ERSS_min_ (or ERSS_max_) or Double Extreme Ranked Set Sample DERSS_min_ (or DERSS_max_). This will complete one cycle. The cycle may be repeated *r* times to obtain a DERSS of size *n = rm*.

This work is the first to discusses the implementation and the performance of the proportional hazards model when the subjects are selected based on the modified ERSS (ERSS_min_ or ERSSmax) and DERSS (DERSS_min_ or DERSSmax) sampling scheme. In this work, we assume that the ranking of subjects, including in the study, is based on one of the available auxiliary variables associated with the variable of interest (time–to–event). Also, we are the first to show theoretically that the proposed sampling schemes improve the Fisher’s information compared with SRS. This implies that Fisher’s information for DERSS is higher than that of ERSS, and ERSS is higher than SRS. The rest of the paper is as follows: Section 2 provides basic survival analysis, definitions, DERSS procedure, and notation. The analysis using the Cox proportional hazards model and its properties using DERSS is discussed in section 3. In Section 4, we provide our simulation study to compare the Cox proportional hazards model’s performance using the modified DERSS to ERSS and SRS. We illustrate the proposed methods using the SEER Incidence Data in section 5. Final remarks are provided in Section 6.

## Preliminaries and notations

### Basic definitions

Let *S*(*t*) denote the survival function at time t with hazard rate function denoted by *h*(*t*) and *f*(*t*) as p.d.f for a random variable of time to an event *T*. Furthermore, survival data is usually not fully observed, and data censoring procedure occurs. The right censoring occurs when the subject has not experienced the event of interest at a given time, which means that we observe only the lower bound for the value of *t* of the censored individuals. On the other hand, type I censoring occurs when the event is unobserved before some pre-specified time, such as at the closing of a study. In this work, we focus on type I right censoring.

### Likelihood function for type I right censored data

In survival data, we define *t*_*i*_ = min(*T*_*i*_, *C*_*i*_)*T*_*i*_ the true survival time and *C*_*i*_ the censoring time for the *i*^*th*^ individual. Let *δ*_*i*_ be an indicator variable, such that

$$\delta_{i}=\left\{\begin{array}{c} 1\;\text{if}\;T=t_{i}\;\text{or}\;T\leq C_{i}\;(\text{uncensored})\\ 0\;\text{if}\;T>t_{i}\;\text{or}\;T>C_{i}\;(\text{right}\;\text{censored}). \end{array}\right.\;$$ (1)

The likelihood function for n observations is given by

$$l(\beta )=\prod_{i=1}^{n}f{\left(t_{i}\right)}^{\delta_{i}}S{\left(t_{i}\right)}^{1-\delta_{i}},$$ (2)

where **β** is the set of parameters of risk factors.

### Sample notation and some basic results of the modified ERSS and DERSS

As in Samawi (2002), let $\left\{X_{ijk}^{l};i=1,2\ldots ,m^{2},j=1,2,\ldots ,m,l=1,2,\ldots ,m,k=1,2,\ldots ,r\right\}$ denotes the *m* independent sets for the kth cycle, each with sample size m^2^ from a p.d.f. f(x) and a distribution function F(x) (absolutely continuous). Using similar notations as in Samawi et al. [21] for selecting DERSSmin, and hence also ERSS_min_, we have, after ranking the sample units within each sample in each set, visually or by any not costly way, we obtain ERSS_min_ as, (X_1(1)*k*_, X_2(1)*k*_,…,X_*m*(1)*k*_, *k* = 1,2,…,*r*) of size m.r. Then DERSSmin is obtained as $Y_{1(1)k}^{}=\text{min}(X_{1(1)k}^{1},X_{2(1)k}^{1},\ldots ,X_{m(1)k}^{1})$, $Y_{2(1)k}^{}=\text{min}(X_{1(1)k}^{2},X_{2(1)k}^{2},\ldots ,X_{m(1)k}^{2})$,…., $Y_{m(1)k}^{}=\text{min}(X_{1(1)k}^{m},X_{2(1)k}^{m},\ldots ,X_{m(1)k}^{m})$. Then *Y*_1(1)*k*_, *Y*_2(1)*k*_,…,*Y*_*m*(1)*k*_ k = 1, 2,…, r, denotes DERSSmin. However, selecting DERSSmax is similar but selecting the maximum instead of the minimum. Samawi et al. [25] showed that the p.d.f of the smallest and the largest order statistics of an i.i.d sample of size m with p.d.f *f*_*X*_(*x*) are respectively given by: *f*_*X*(1)_(*x*) = *m*(1−*F*_*X*_(*x*))^*m*−1^ *f*_*X*_(*x*) and *f*_*X*(*m*)_(*x*) = *m*(*F*_*X*_(*x*))^*m*−1^ *f*_*X*_(*x*) Also, let *Y*_*i*(1)*k*_ have p.d.f *g*_(1)_(*y*) and c.d.f *G*_(1)_(*x*) and *Y*_*i*(*m*)*k*_ have p.d.f *g*_(m)_(*y*) and c.d.f *G*_(m)_(*y*) where i = 1,2,…,m and k = 1, 2,…, r., then

1. $G_{Y(1)}(y)=1-{\left[1-F_{X(1)}(y)\right]}^{m}=1-{\left[1-F_{X}(y)\right]}^{m^{2}}$,
2.
${\text{g}}_{\text{Y(1)}}=mf_{X(1)}(y){\left[1-F_{X(1)}(y)\right]}^{m-1}{=\text{m}}^{2}\;f_{X}(y)\;[1-F_{Y}(y){\text{]}}^{m^{2}-1}$

3.
$G_{Y(m)}(y)={\left[F_{X}(y)\right]}^{m^{2}}\;$

4.
${\text{g}}_{\text{Y(m)}}={\text{m}}^{2}\;f_{X}(y)\;[F_{X}(y){\text{]}}^{m^{2}-1}$

### Cox proportional hazards model using ERSS_min_ and DERSS_min_

This section investigates the Cox Proportional Hazards Model using ERSS_min_ and DERSS_min_ when the survival time and the ranked auxiliary variable (X) are positively correlated. When they have a negative correlation, we can use either ERRS_max_ or DERSS_max_, and all consequence results can be derived in similar manners. We will focus on DERSS_min_ in our consequence discussion.

For the k^th^ cycle, *k* = 1,2,…,*r*, let *Y*_1(1)*k*_,*Y*_*2*(1)*k*_,…,*Y*_*m*(1)*k*_ be the measurements obtained on the auxiliary variable, using DERSS_min_ with size *n* = *rm*. Moreover, let the vector *V*_*i*[1]*k*_ = (*X*_*i*[1]*k*_…,*X*_*i*[1]*kp*_, *Y*_*i*(1)*k*_)′, *i* = 1,2,…,*m*;*k* = 1,2,…,*r* represent the *p+1* explanatory variables’ observations and one auxiliary variable obtained from the sampled unit in the *ith* set and *kth* cycle. Note that the notation (.) stands for perfect ranking while the notation [.] stands for imperfect ranking.

The partial likelihood is usually used to estimate the model parameters **β** = (*β*_1_, *β*_2_,…,*β*_*p*+1_)′ in the Cox model. Under SRS assumptions, Cox and Oakes [26] showed that the estimators of **β** = (*β*_1_, *β*_2_,…,*β*_*p*+1_)′ from partial likelihood are consistent and asymptotically normal distributed. However, Cox and Oakes [26] showed a loss of precision in estimation by using partial likelihood compared to the full likelihood. Also, they indicated that the sample size and the proportion of censoring observation affect inference precision. When the percentage of censoring increases and the sample size is small, the estimators from partial likelihood lose more precision. We propose to use DERSS to improve the accuracy of inferences based on the partial likelihood estimation of Cox model parameters.

### Partial likelihood for the Cox model

To estimate the parameter in the Cox proportional hazards model, we order the random sample of size *n* individuals according to the rank of their survival times, *t*_1_ < *t*_2_ <… < *t*_*n*_, assuming there are no ties for the observations. Instead of using the full likelihood function in (5), Cox [27] proposed partial likelihood as follows,

$$\frac{\text{exp}(v_{i[1]k}\beta )}{\sum_{h\in R(t_{i[1]k})}^{}\text{exp}(v_{h}\beta )}.$$ (3)

It is the consequence of the conditional probability that an individual experiences an event at time *t*_*i*[1]*k*_, (*i* = 1,2,…,*m*;*k* = 1,2,…,*r*) given that the individual in the event’s risk set at *t*_*i*[1]*k*_, where *R*(*t*_*i*[1]*k*_) be the set of all individuals who have not experienced the event and are uncensored just prior to *t*_*i*[1]*k*_. Under DERSS_min_, and as described by Samawi et al. [21] the partial likelihood function (PLHF) can be written as

$$l(\beta )=\prod_{i=1}^{m}\prod_{k=1}^{r}{\left[\frac{\text{exp}(v_{i[1]k}\beta )}{\sum_{h\in R(t_{i[1]k})}^{}\text{exp}(v_{h}\beta )}\right]}^{\delta_{i[1]k}}.$$ (4)

When the observation is censored, the product of the conditional probabilities is 1 and equivalently can be written as

$$l(\beta )=\prod_{i=1}^{m}\prod_{k=1}^{r_{d}}{\left[\frac{\text{exp}(v_{i[1]k}\beta )}{\sum_{h\in R(t_{i[1]k})}^{}\text{exp}(v_{h}\beta )}\right]}^{},$$ (5)

where (*r*_*d*_.*m*) is the number of events (failed subjects) in a sample of size (*n* = *r*.*m*). The log-likelihood can be written as,

$$L(\beta )=\text{log}[l(\beta )]=\sum_{i=1}^{m}\sum_{k=1}^{r_{d}}\left\{v_{i[1]k}^{\prime }\beta -\text{log}\left(\sum_{h\in R(t_{i[1]k})}^{}\text{exp}(v_{h}^{\prime }\beta ))\right)\right\}.$$ (6)

Since the partial likelihood function depends on the ordering of survival times and then the baseline hazard distribution is inherent in ordering the survival times, and no shape is described. Therefore, the produced partial likelihood is only based on the uncensored observations, and hence this may result in a large sample size requirement. However, in contrast to SRS, ERSS and DERSS naturally provide a larger percentage of events, as indicated by Samawi et al. [21, 25] and may result in requiring a smaller sample size. For estimating the j^th^ parameter (*β*_*j*_), we need to solve the following equation

$$\frac{dL(\beta )}{d\beta_{j}}=\sum_{i=1}^{m}\sum_{k=1}^{r_{d}}\left\{x_{i[1]kj}-\sum_{h\in R(t_{i[1]k})}^{}x_{hj}g_{h}(y_{i(1)k};R(t_{i[1]k}))\right\}=0,$$ (7)

where $g_{h}(y_{i(1)k};R(t_{i[1]k}))=\frac{\text{exp}(v_{h}^{\prime }\beta )}{\sum_{h\in R(t_{i[1:]k})}^{}\text{exp}(v_{h}^{\prime }\beta )}$ is the weight of the *h*^*th*^ failed subject because $\sum_{h\in R(t_{i[1]k})}^{}g_{h}(y_{i(1)k};R(t_{i[1]k}))=1$. Clearly, the MLE ($\hat{\beta }$)is the solution of Eq (7).

For the consistency and the asymptotic properties of $\hat{\beta }$, see [26].

To show the superiority of using ERSS and DERSS proposed in this paper, we need to derive the Fisher’s information matrix of $\hat{\beta }$, say *I*(**β**). To find *I*(**β**) we take the second derivative of *L*(**β**):

$$I{\left(\beta \right)}_{DERSS_{\text{min}}}=-E_{V_{\left(1\right)}}E_{t_{\left[1\right]}|V_{\left(1\right)}}{\left(\frac{\partial^{2}L(\hat{\beta })}{\partial {\hat{\beta }}^{2}}\right)}_{\left(P+1)x(P+1\right)}.$$

For the *jth* covariate, we have

$$-\left(\frac{\partial^{2}L(\beta )}{\partial \beta_{j}^{2}}\right)=\sum_{i=1}^{m}\sum_{k=1}^{r_{d}}\left({\sum_{h\in R(t_{i[1]k})}^{}\left[x_{hj}^{}-\left(\sum_{h\in R(t_{i[1]k})}^{}x_{hj}^{}g_{h}(y_{\left(1\right)};R(t_{i[1]k}))\right)\right]}^{2}g_{h}(y_{\left(1\right)};R(t_{i[1]k}))\right),$$ (8)

which is a function of the ranked auxiliary covariate (Y) used to select subjects from the population using the DERSS_min_ scheme randomly and assumed to have some distribution *F*_*Y*_(*y*), with a continuous density *f*_*Y*_(*y*). As suggested by [21, 25] ranking on this variable induces a corresponding ordering on the response variable (*T*), which leads to improved precision. When using DERSS_min_, we have taken the minimum judgment ranking from each set of size m, and as shown by [25], the density function of *Y*_(1)_ is ${\text{g}}_{Y(1)}(y)={\text{m}}^{2}\;f_{Y}(y)\;[1-F_{Y}(y){\text{]}}^{m^{2}-1}$. Then the Fisher information using DERSS_min_ is given by:

$$\begin{array}{l} I{\left(\beta_{j}\right)}_{DERSS_{\text{min}}}=-E_{Y_{\left(1\right)}}E_{t_{\left[1\right]}|Y_{\left(1:\right)}}\left(\frac{\partial^{2}L(\beta )}{\partial \beta_{j}^{2}}\right)\\ =E_{Y_{\left(1\right)}}\left(\sum_{i=1}^{m}\sum_{k=1}^{r_{d}}\left({\sum_{h\in R(t_{i[1]k})}^{}\left[x_{hj}^{}-\left(\sum_{h\in R(t_{[1:j]i})}^{}x_{hj}^{}g_{h}(Y_{\left(1\right)};R(t_{[1:j]i}))\right)\right]}^{2}g_{h}(Y_{\left(1\right)};R(t_{i[1]k}))\right)\right)\\ =r_{d}\sum_{i=1}^{m}\int_{v}\left({\sum_{h\in R(t_{i[1]k})}^{}\left[x_{hj}^{}-\left(\sum_{h\in R(t_{i[1]k})}^{}x_{hj}^{}g_{h}(Y_{\left(1\right)};R(t_{i[1]k}))\right)\right]}^{2}g_{h}(Y_{\left(1\right)};R(t_{i[1]k}))\right)mf_{Y_{\left(1\right)}}(y){\left(1-F_{Y(1)}(y)\right)}^{j-1}dy. \end{array}$$

Since we assume that *β*_*P*+1_ > 0, then the number of events and *R*(*t*_*i*[1]*k*_) will be increased. This implies that *g*_*h*_(*y*_(1)_; *R*(*t*_*i*[1]*k*_)) is a decreasing function of *y*. Also, $m{\left(1-F_{Y_{\left(1\right)}}(y)\right)}^{m-1}$ is a decreasing function of *y*, thus as in See and Chen [28]

$$\begin{array}{l} I{\left(\beta_{j}\right)}_{DERSS_{\text{min}}}\\ \geq \left\{r_{d}\sum_{i=1}^{m}\int_{v}\left({\sum_{h\in R(t_{i[1]k})}^{}\left[x_{hj}^{}-\left(\sum_{h\in R(t_{i[1]k})}^{}x_{hj}^{}g_{h}(Y_{\left(1\right)};R(t_{i[1]k}))\right)\right]}^{2}g_{h}(Y_{\left(1\right)};R(t_{i[1]k}))\right)f_{Y_{\left(1\right)}}(y)dy\right\}\\ \left\{\int_{v}mf_{Y_{\left(1\right)}}(y){\left(1-F_{Y_{\left(1\right)}}(y)\right)}^{m-1}dy\right\} \end{array}$$

However, $\int_{v}mf_{Y_{\left(1\right)}}(y){\left(1-F_{Y_{\left(1\right)}}(y)\right)}^{m-1}dy=1$, therefore,

$$\begin{array}{l} I{\left(\beta_{j}\right)}_{DERSS_{\text{min}}}\geq r_{d}\sum_{i=1}^{m}\int_{v}\left({\sum_{h\in R(t_{i[1]k})}^{}\left[x_{hj}^{}-\left(\sum_{h\in R(t_{i[1]k})}^{}x_{hj}^{}g_{h}(V_{\left(1\right)};R(t_{i[1]k}))\right)\right]}^{2}g_{h}(V_{\left(1\right)};R(t_{i[1]k}))\right)f_{Z_{\left(1\right)}}(v)dv=I{\left(\beta_{j}\right)}_{ERSS_{\text{min}}}\\ \geq I{\left(\beta_{j}\right)}_{SRS} \end{array}$$ (9)

Hence, the last inequality in [15] can be argued similarly. These inequalities are still held when the correlation between the survival time and Y is negative, and DERSS_max_ is used. The inequalities in [15] show that using DERSS_min_ provides more information than ERSS and ERSS provides more information than SRS, and hence DERSS_min_ may require a smaller sample size to achieve similar precision as for ERSS or SRS.

## Simulation study and results

For comparison purposes, we used simulation to evaluate the Cox proportional hazards model’s performance when ERSSmin and DERSSmin are used. We assume that the Ranked Auxiliary Covariate is positively correlated with survival time. We compared DERSSmin with both ERSSmin and SRS. The power of testing the hypothesis of no treatment effect after controlling for the auxiliary variables is compared. We also studied the performance of estimating the conditional hazard ratios for risk factors and their confidence intervals. We used different conditional hazard ratios and different values of the association between survival time and the auxiliary variables. Two combinations of sample sizes of *m* and *r* are considered used in the simulations, i.e., (m = 20, r = 10), and (m = 30, r = 20). We used 5000 simulated samples in our simulation.

The parameter *β*_*1*_ is the association between T and Y, and *β*_*2*_ is the risk factor parameter. The simulation results are given in Tables 1–3 for a dichotomous risk factor, while the results of a continuous risk factor are presented in Tables 4–6. We estimated the empirical nominal value (*α* = 0.05) and the tests’ power (see Tables 1 and 4). The estimated conditional hazard ratios and their MSEs are reported in Tables 2 and 5, while the 95% confidence intervals for hazard ratios are reported in Tables 3 and 6.

**Table 1:** Evaluating the (*α* = 0.05) and the power of testing *H*_*o*_: *β*_2_ = 0 vs *H*_*a*_: *β*_2_ ≠ 0 adjusting for (Z) in the model. (Binary risk factor) {Censoring variable = ci = U(0,1)*1.5}.

Parameters		*m = 20 and r = 10*					
*β*_1_	*β*_2_	DERSS_min_		ERSS_min_		SRS	
		Events %	Power of the test	Events %	Power of the test	Events %	Power of the test
0.2	0.0	0.730	0.045	0.674	0.057	0.567	0.048
0.2	0.3	0.762	0.416	0.710	0.396	0.608	0.333
0.2	0.5	0.781	0.843	0.732	0.825	0.635	0.786
0.2	0.8	0.805	1.000	0.761	1.000	0.672	0.999
0.5	0.0	0.885	0.048	0.801	0.046	0.566	0.045
0.5	0.3	0.901	0.513	0.825	0.487	0.606	0.366
0.5	0.5	0.908	0.906	0.839	0.888	0.630	0.787
0.5	0.8	0.916	1.000	0.854	1.000	0.662	0.995
1.0	0.0	0.973	0.052	0.913	0.061	0.557	0.059
1.0	0.3	0.976	0.530	0.924	0.507	0.590	0.363
1.0	0.5	0.977	0.921	0.928	0.907	0.612	0.759
1.0	0.8	0.980	1.000	0.935	1.000	0.642	0.991
m = 30 and r = 20							
0.2	0.0	0.743	0.046	0.684	0.048	0.566	0.042
0.2	0.3	0.775	0.889	0.721	0.868	0.611	0.811
0.2	0.5	0.791	0.999	0.741	1.000	0.636	0.998
0.2	0.8	0.812	1.000	0.767	1.000	0.671	1.000
0.5	0.0	0.898	0.055	0.816	0.057	0.564	0.054
0.5	0.3	0.911	0.928	0.840	0.921	0.605	0.803
0.5	0.5	0.919	1.000	0.853	1.000	0.630	0.997
0.5	0.8	0.926	1.000	0.867	1.000	0.663	1.000
1.0	0.0	0.978	0.049	0.927	0.047	0.555	0.044
1.0	0.3	0.982	0.947	0.937	0.945	0.589	0.805
1.0	0.5	0.983	1.000	0.941	1.000	0.611	0.998
1.0	0.8	0.984	1.000	0.947	1.000	0.641	1.000

**Table 2:** Estimation of Hazard ratio (HR) estimation and their MSE (Binary risk factor) {Censoring variable = ci = U(0,1)*1.5}.

m = 20 and r = 10							
		DERSS_min_		ERSS_min_		SRS	
*β*_1_	HR	Estimate	MSE	Estimate	MSE	Estimate	MSE
0.2	1.000	1.009	0.030	1.008	0.033	1.007	0.039
0.2	1.350	1.360	0.052	1.36	0.057	1.359	0.066
0.2	1.649	1.672	0.083	1.679	0.089	1.690	0.108
0.2	2.226	2.258	0.148	2.256	0.152	2.257	0.170
0.5	1.000	1.018	0.023	1.018	0.026	1.023	0.04
0.5	1.350	1.373	0.045	1.376	0.049	1.381	0.073
0.5	1.649	1.683	0.075	1.684	0.081	1.688	0.108
0.5	2.226	2.271	0.143	2.268	0.151	2.289	0.186
1.0	1.000	1.012	0.021	1.012	0.024	1.011	0.038
1.0	1.350	1.373	0.045	1.371	0.047	1.370	0.072
1.0	1.649	1.671	0.067	1.673	0.072	1.681	0.106
1.0	2.226	2.273	0.14	2.277	0.147	2.275	0.200
m = 30 and r = 20							
0.2	1.000	1	0.009	1	0.009	1.003	0.012
0.2	1.350	1.353	0.015	1.354	0.016	1.355	0.019
0.2	1.649	1.653	0.023	1.653	0.025	1.653	0.029
0.2	2.226	2.231	0.042	2.232	0.045	2.231	0.051
0.5	1.000	1.006	0.008	1.008	0.009	1.008	0.012
0.5	1.350	1.359	0.016	1.358	0.016	1.359	0.022
0.5	1.649	1.654	0.022	1.655	0.023	1.656	0.031
0.5	2.226	2.241	0.041	2.24	0.043	2.241	0.055
1.0	1.000	1.001	0.007	1.001	0.007	1.004	0.012
1.0	1.350	1.356	0.013	1.357	0.014	1.359	0.021
1.0	1.649	1.66	0.02	1.661	0.02	1.659	0.031
1.0	2.226	2.238	0.044	2.237	0.045	2.238	0.059

**Table 3:** Estimating 95% confidence Interval length and coverage probability (CP) of the Hazard Ratio (HR) (Binary risk factor) {Censoring variable = ci = U(0,1)*1.5}.

		DERSS_min_		ERSS_min_		SRS	
m = 20 and r = 10							
*β*_1_	HR	length	CP	length	CP	Length	CP
0.2	1.000	0.677	0.955	0.705	0.943	0.771	0.952
0.2	1.350	0.899	0.959	0.932	0.958	1.008	0.948
0.2	1.649	1.107	0.944	1.147	0.948	1.242	0.944
0.2	2.226	1.514	0.951	1.550	0.953	1.644	0.958
0.5	1.000	0.620	0.952	0.652	0.954	0.783	0.955
0.5	1.350	0.837	0.954	0.875	0.951	1.029	0.947
0.5	1.649	1.038	0.948	1.078	0.943	1.245	0.942
0.5	2.226	1.445	0.945	1.484	0.943	1.685	0.957
1.0	1.000	0.588	0.948	0.608	0.939	0.784	0.941
1.0	1.350	0.804	0.943	0.825	0.946	1.037	0.947
1.0	1.649	0.994	0.953	1.019	0.956	1.261	0.949
1.0	2.226	1.403	0.943	1.431	0.948	1.703	0.939
*m = 30 and r = 20*							
0.2	1.000	0.376	0.954	0.392	0.952	0.432	0.958
0.2	1.350	0.501	0.956	0.519	0.961	0.564	0.963
0.2	1.649	0.612	0.953	0.631	0.946	0.681	0.953
0.2	2.226	0.839	0.956	0.860	0.963	0.914	0.958
0.5	1.000	0.344	0.945	0.361	0.943	0.435	0.946
0.5	1.350	0.465	0.926	0.484	0.935	0.569	0.948
0.5	1.649	0.572	0.953	0.592	0.949	0.686	0.948
0.5	2.226	0.800	0.955	0.820	0.954	0.924	0.949
1.0	1.000	0.328	0.951	0.337	0.953	0.437	0.956
1.0	1.350	0.447	0.952	0.458	0.954	0.578	0.951
1.0	1.649	0.556	0.953	0.567	0.955	0.698	0.950
1.0	2.226	0.778	0.933	0.788	0.937	0.940	0.940

**Table 4:** Evaluating (*α* = 0.05) and the power of testing *H*_*o*_: *β*_2_ = 0 vs *H*_*a*_: *β*_2_ ≠ 0 adjusting for the auxiliary variable (Z) in the model. (Continuous risk factor) {Censoring variable = ci = U(0,1)*1.5}.

Parameters		*m = 20 and r = 10*					
*β*_1_	*β*_2_	DERSS_min_		ERSS_min_		SRS	
		Events %	Power of the test	Events %	Power of the test	Events %	Power of the test
0.2	0.0	0.731	0.057	0.675	0.047	0.568	0.045
0.2	0.3	0.726	0.936	0.670	0.924	0.565	0.884
0.2	0.5	0.715	1.000	0.662	1.000	0.565	1.000
0.2	0.8	0.699	1.000	0.650	1.000	0.559	1.000
0.5	0.0	0.885	0.058	0.799	0.050	0.564	0.059
0.5	0.3	0.879	0.973	0.793	0.960	0.562	0.866
0.5	0.5	0.870	1.000	0.783	1.000	0.563	0.999
0.5	0.8	0.852	1.000	0.764	1.000	0.559	1.000
1.0	0.0	0.972	0.047	0.912	0.048	0.556	0.057
1.0	0.3	0.971	0.985	0.908	0.977	0.557	0.861
1.0	0.5	0.969	1.000	0.902	1.000	0.556	0.998
1.0	0.8	0.963	1.000	0.887	1.000	0.553	1.000
*m = 30 and r = 20*							
0.2	0.0	0.743	0.046	0.683	0.053	0.567	0.053
0.2	0.3	0.736	1.000	0.678	1.000	0.566	1.000
0.2	0.5	0.726	1.000	0.671	1.000	0.564	1.000
0.2	0.8	0.707	1.000	0.656	1.000	0.559	1.000
0.5	0.0	0.898	0.049	0.816	0.046	0.564	0.047
0.5	0.3	0.893	1.000	0.810	1.000	0.563	1.000
0.5	0.5	0.885	1.000	0.799	1.000	0.561	1.000
0.5	0.8	0.867	1.000	0.779	1.000	0.557	1.000
1.0	0.0	0.978	0.051	0.927	0.050	0.556	0.043
1.0	0.3	0.978	1.000	0.925	1.000	0.558	1.000
1.0	0.5	0.976	1.000	0.918	1.000	0.556	1.000
1.0	0.8	0.970	1.000	0.904	1.000	0.551	1.000

**Table 5:** Estimating Hazard ratio (HR) estimation and their MSE (Continuous risk factor) {Censoring variable = ci = U(0,1)*1.5}.

m = 20 and r = 10							
		DERSS_min_		ERSS_min_		SRS	
*β*_1_	HR	Estimate	MSE	Estimate	MSE	Estimate	MSE
0.2	1.000	0.998	0.007	0.999	0.008	1.000	0.009
0.2	1.350	1.366	0.015	1.364	0.016	1.366	0.020
0.2	1.649	1.680	0.026	1.679	0.027	1.678	0.029
0.2	2.226	2.257	0.054	2.255	0.056	2.261	0.063
0.5	1.000	1.002	0.006	1.003	0.007	1.008	0.011
0.5	1.350	1.358	0.012	1.358	0.013	1.360	0.018
0.5	1.649	1.663	0.020	1.663	0.022	1.666	0.031
0.5	2.226	2.255	0.047	2.260	0.053	2.268	0.069
1.0	1.000	1.004	0.006	1.006	0.006	1.006	0.010
1.0	1.350	1.362	0.011	1.362	0.011	1.362	0.017
1.0	1.649	1.659	0.020	1.661	0.021	1.668	0.033
1.0	2.226	2.258	0.041	2.259	0.043	2.273	0.068
m = 30 and r = 20							
0.2	1.000	1.000	0.002	1.000	0.003	1.001	0.003
0.2	1.350	1.354	0.004	1.354	0.005	1.355	0.005
0.2	1.649	1.655	0.007	1.654	0.007	1.657	0.009
0.2	2.226	2.241	0.017	2.241	0.019	2.245	0.021
0.5	1.000	1.001	0.002	1.002	0.002	1.000	0.003
0.5	1.350	1.351	0.004	1.351	0.004	1.351	0.006
0.5	1.649	1.654	0.006	1.654	0.007	1.654	0.009
0.5	2.226	2.230	0.014	2.232	0.015	2.236	0.020
1.0	1.000	1.001	0.002	1.000	0.002	0.999	0.003
1.0	1.350	1.351	0.003	1.351	0.004	1.354	0.006
1.0	1.649	1.659	0.006	1.660	0.006	1.660	0.010
1.0	2.226	2.234	0.012	2.234	0.013	2.234	0.020

**Table 6:** Estimating 95% confidence interval length and coverage probability (CP) of the Hazard Ratio (HR) (Continuous risk factor) {Censoring variable = ci = U(0,1)*1.5}.

		DERSS_min_		ERSS_min_		SRS	
m = 20 and r = 10							
*β*_1_	HR	length	CP	length	CP	length	CP
0.2	1.000	0.333	0.943	0.346	0.953	0.378	0.955
0.2	1.350	0.471	0.947	0.489	0.944	0.531	0.945
0.2	1.649	0.613	0.948	0.634	0.956	0.680	0.960
0.2	2.226	0.908	0.951	0.935	0.954	0.998	0.955
0.5	1.000	0.305	0.942	0.320	0.950	0.383	0.941
0.5	1.350	0.429	0.953	0.449	0.957	0.530	0.959
0.5	1.649	0.555	0.948	0.580	0.949	0.675	0.945
0.5	2.226	0.840	0.960	0.878	0.956	1.003	0.951
1.0	1.000	0.291	0.953	0.301	0.952	0.387	0.943
1.0	1.350	0.410	0.960	0.422	0.961	0.532	0.967
1.0	1.649	0.531	0.953	0.546	0.946	0.682	0.953
1.0	2.226	0.803	0.950	0.827	0.952	1.013	0.950
m = 30 and r = 20							
0.2	1.000	0.188	0.954	0.195	0.947	0.215	0.947
0.2	1.350	0.262	0.955	0.272	0.954	0.297	0.954
0.2	1.649	0.338	0.956	0.349	0.956	0.378	0.964
0.2	2.226	0.506	0.942	0.521	0.949	0.557	0.940
0.5	1.000	0.171	0.951	0.179	0.954	0.215	0.953
0.5	1.350	0.239	0.953	0.250	0.945	0.297	0.949
0.5	1.649	0.310	0.960	0.323	0.954	0.377	0.949
0.5	2.226	0.464	0.946	0.484	0.952	0.554	0.954
1.0	1.000	0.164	0.949	0.168	0.950	0.217	0.957
1.0	1.350	0.230	0.948	0.235	0.950	0.300	0.957
1.0	1.649	0.299	0.950	0.306	0.954	0.382	0.949
1.0	2.226	0.446	0.948	0.457	0.951	0.556	0.947

From Table 1, we can conclude that, when the risk factor is dichotomous, testing the hypothesis of the risk factor’s effect, controlling for the Ranked Auxiliary Covariate Y in the model, the DERSS_min_ results in a more powerful test than SRS and ERSS_min_. All SRS, ERSS_min_, and DERSS_min_ achieved the test nominal value (0.05) under the null hypothesis in all cases. We demonstrated the test’s power increases as the set size *m* increase and/or the value of *β*_1_ increases. However, using DERSS_min_ in the Cox proportional hazard model provides greater power than SRS and ERSS_min_, in all cases. Similarly, from Tables 2 and 3, DERSS_min_ has smaller MSE and narrower confidence intervals in estimating the hazard ratios. Similar results are concluded from Tables 4–6 when the risk factor is a continuous variable.

## Application using the SEER incidents data set

We illustrate the Cox proportional hazards model based on DERSS, ERSS, and SRS using SEER Incident Data [29]. We selected histologic grade as the primary predictor of interest and month of life after a breast cancer diagnosis as the outcome. Due to breast cancer, deaths were considered death and being alive, or Deaths due to other causes deemed to be censored. In the Cox proportional hazards model, we also include age and tumor marker1 as confounders. The data included 454,517 individuals, and 233,125 have complete data for a grade (1: well-differentiated; 2: moderately differentiated; 3: poorly differentiated; 4: undifferentiated). Our analysis was based on complete data. Since grade levels 3 and 4 are crossed on the survival plots, which violates the proportional hazards assumption for the Cox model, we combined grade 3 and grade 4 into one single group as poorly differentiated or undifferentiated and denoted the new grade variable (1: well-differentiated; 2: moderately differentiated; 3: poorly differentiated or undifferentiated).

We treated the entire data set of 233,125 observations as a population and randomly selected ERSS and DERSS samples (with m = 15, r = 20). We selected ERSS and DERSS based on ranking on the lifetime directly since most of the data set variables are categorical. The only potential continuous variable for ranking is age, which is only weakly associated with lifetime with hazards ratio close to 1. We performed the hypothesis testing of no difference in survival time between the patients with a different histologic grade at diagnosis, adjusting for the covariate age. Table 7 represents the survival analysis results for a Cox proportional hazards model using all available data. The estimation of parameters and the conditional hazard ratios are treated as actual for comparison purposes in this example.

**Table 7:** Variables in the Equation using all the data (n = 233,125).

Variables	B	SE	Wald	Sig.	Exp(B)
Age	0.0040	0.0004	10.66	<0.0001	1.0040
Grade 2 vs 1	0.9889	0.0277	35.73	<0.0001	2.6882
Grade (3 or 4) vs 1	1.8042	0.0267	67.55	<0.0001	6.0752

**Fig 1:**
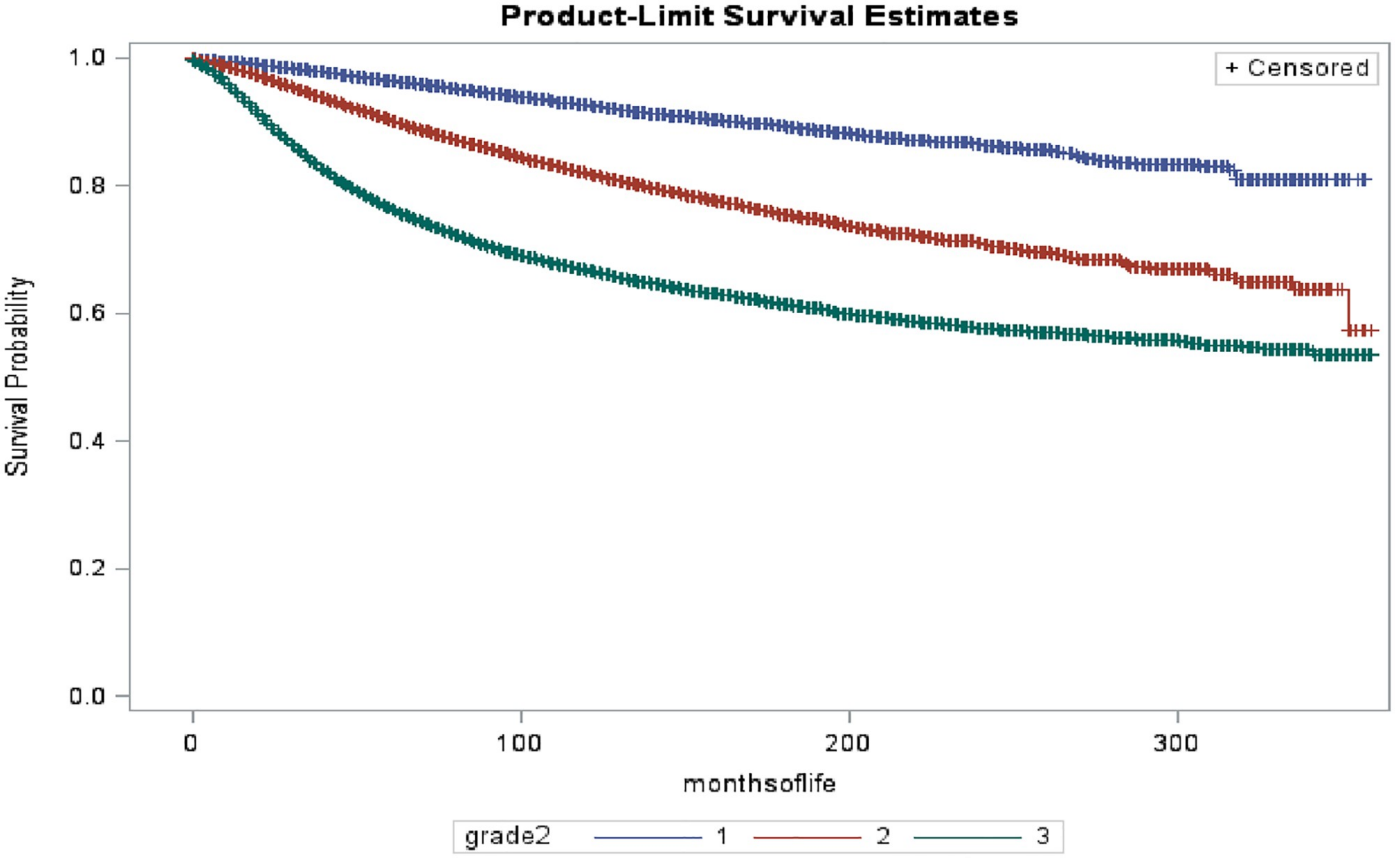
Survival function for the grades (1: Well-differentiated; 2: Moderately differentiated; 3: Poorly differentiated or undifferentiated) using all complete data (n = 233,125).

Table 7 and Fig 1 are the results of the survival analysis based on the entire data. Fig 1 indicates that the proportional hazard assumption of the model is valid. Also, Fig 1 shows that the survival chance for grade 1 is higher than grade2 and grade 3, and grade 2 is higher than grade 3 of the disease. However, based on the p-values, the test of the effect of grade 3 on survival time compared with grade 1 and 2 was significant, controlling for age, based on the 500 bootstrap samples of SRS, ERSS, and DERSS of size n = 300. Both ERSS and DERSS used survival time for ranking and chose the samples with the minimum survival time. The DERSS samples give smaller p-values for the majority of the time and coincide with the whole data analysis. The estimated power out of the 5000 runs was 0.944, 0.930, and 0.856, respectively, under DERSS, ERSS, and SRS. Therefore, whenever possible, ERSS and/or DERSS are to be recommended for survival analysis.

## Final remarks and conclusion

It is essential in collecting data to have a sampling scheme that is cost-effective and less time-consuming. ERSS and DERSS, which are RSS modifications, are encouraging sampling techniques that can effectively have more efficient estimators with less cost than SRS. We proposed a more efficient survival regression analysis method based on Double Extreme Ranked Set Sampling (DERSS) and Extreme Ranked Set Sampling (ERSS). Subjects are assumed to be ranked based on an auxiliary variable associated with the response variable. We discussed parameter estimation based on the maximum likelihood approach and provided an expression for the estimated variance based on the inverse information matrix. Also, we discussed the asymptotic behavior of the ML estimators. Our findings conclude that using ERSS and DERSS can significantly increase power when used in a Cox proportional hazards model. We show that the test’s power increases as the set size *m* increases through the simulation studies. Moreover, ERSS and DERSS provide more efficient estimates of the parameters associated with hazard ratios in smaller MSEs and narrower confidence intervals than those under SRS.
